# All-Trans Retinoic Acid Activity in Acute Myeloid Leukemia: Role of Cytochrome P450 Enzyme Expression by the Microenvironment

**DOI:** 10.1371/journal.pone.0127790

**Published:** 2015-06-05

**Authors:** Meng Su, Salvador Alonso, Jace W. Jones, Jianshi Yu, Maureen A. Kane, Richard J. Jones, Gabriel Ghiaur

**Affiliations:** 1 Sidney Kimmel Comprehensive Cancer Center, Johns Hopkins University, Baltimore, Maryland, United States of America; 2 University of Maryland School of Pharmacy, Baltimore, Maryland, United States of America; Roswell Park Cancer Institute, UNITED STATES

## Abstract

Differentiation therapy with all-trans retinoic acid (atRA) has markedly improved outcome in acute promyelocytic leukemia (APL) but has had little clinical impact in other AML sub-types. Cell intrinsic mechanisms of resistance have been previously reported, yet the majority of AML blasts are sensitive to atRA *in vitro*. Even in APL, single agent atRA induces remission without cure. The microenvironment expression of cytochrome P450 (CYP)26, a retinoid-metabolizing enzyme was shown to determine normal hematopoietic stem cell fate. Accordingly, we hypothesized that the bone marrow (BM) microenvironment is responsible for difference between *in vitro* sensitivity and *in vivo* resistance of AML to atRA-induced differentiation. We observed that the pro-differentiation effects of atRA on APL and non-APL AML cells as well as on leukemia stem cells from clinical specimens were blocked by BM stroma. In addition, BM stroma produced a precipitous drop in atRA levels. Inhibition of CYP26 rescued atRA levels and AML cell sensitivity in the presence of stroma. Our data suggest that stromal CYP26 activity creates retinoid low sanctuaries in the BM that protect AML cells from systemic atRA therapy. Inhibition of CYP26 provides new opportunities to expand the clinical activity of atRA in both APL and non-APL AML.

## Introduction

Acute myeloid leukemia (AML) is characterized by impaired differentiation and uncontrolled proliferation with subsequent accumulation of immature cells (blasts). Even though the treatment results in AML have improved over the past 30 years, more than 50% of young adults and 90% of older patients die of their disease[1]. Advances in the treatment of one AML subtype, acute promyelocytic leukemia (APL), raised hopes that all-trans retinoic acid (atRA)-based therapies might improve outcomes in other AML subtypes. In APL, the C-terminus of retinoic acid receptor α (RARα) on chromosome 17 is most often fused with N-terminus of promyelocytic leukemia protein (PML) on chromosome 15[2]. The resultant fusion protein, PML-RARα has a dominant negative effect on retinoic acid signaling and blocks differentiation by recruiting abnormal transcription factors and histone-modifying enzymes to critical genes. atRA when used at pharmacological concentrations is able to bind PML-RARα and overcome its inhibitory effects, thus allowing transcription of target genes. APL progenitors exposed to atRA *in vitro* or during clinical treatment will continue their differentiation program into neutrophils which eventually senesce.

The introduction of atRA in western medicine clinical protocols in 1980’s changed the face of APL from one of the most malignant types of AML to the most curable[3]. Although the PML-RARα translocation appears to enhance the sensitivity of APL to atRA and several intrinsic mechanisms of atRA resistance have been identified, including overexpression of Tal1, expression of PRAME as well as epigenetic silencing or mutation of RARα [4–7], the majority of non-APL AMLs and even other cancers remain sensitive to atRA *in vitro[7–12]*. Several clinical trials have even suggested a clinical benefit for atRA in at least some subtypes of AML[13–15], although most trials have not confirmed these results[16–18]. It is unclear why atRA has activity against non-APL AML *in vitro*, but limited clinical activity. Moreover, atRA as a single agent complete remissions (CRs) in APL patients, but all patients eventually relapse[19] with a median duration of CR of about 5 months[20]. Thus even in APL there exists minimal residual disease (MRD) that remains resistant to atRA therapy[19]. While combinations of atRA with chemotherapy or arsenic trioxide eliminates MRD in APL and produces cures[21], understanding the mechanism responsible for persistence of MRD in APL patients treated with atRA monotherapy may have important implications for expanding atRA-based therapies to non-APL AML.

RA’s precursor, vitamin A (retinol), plays unique roles in mammalian ontogeny and homeostasis across multiple cellular systems[22]. Since both RA deficiency as well as excess has deleterious effects, some incompatible with life, organisms have developed feedback mechanisms to control retinoid levels. Thus, tissue levels of RA reflect the balance between biosynthesis from vitamin A and inactivation, mostly via cytochrome P450 (CYP) 26 family. While hepatic CYP26 plays an important role in maintaining systemic retinoid homeostasis[23], recent reports have also implicated these enzymes in local control of RA signaling in the microenvironment. In fetal gonads, Sertoli cell expression of CYP26B1 determines the fate of the germ cells through modulating atRA bioavailability[24]. We recently found that the bone marrow (BM) microenvironment similarly expresses CYP26, which protects human hematopoietic stem cells (HSCs) from physiological retinoid-induced differentiation and promotes their self-renewal[25].

Here we evaluate if stromal CYP26 also protects leukemia cells from pharmacological levels of atRA. We found that BM stroma degraded pharmacological concentrations of atRA, rendering even sensitive APL cells resistant to atRA. Moreover, non-APL leukemia cells were also highly sensitive to atRA treatment in the absence of BM stroma, but became resistant in stromal co-culture conditions; inhibition of CYP26 reversed the stromal-mediated atRA resistance.

## Materials and Methods

### Cell lines

The human APL cell line NB4[26] was cultured in RPMI 1640 (Gibco, Rockville, MD, USA) with 2 mM L-glutamine (Life Technologies), 100 μg/mL penicillin-streptomycin (Gibco), and 10% fetal calf serum (FCS) (Sigma-Aldrich). The M2 AML cells Kasumi-1[27] were cultured in RPMI 1640 + 20%FCS and the NPM1 mutated OCI/AML3 cells were cultured in minimum essential media (α-MEM) (Corning Cellgro) with 2 mM L-glutamine, 50 μg/mL penicillin-streptomycin, and 20% FCS. The mouse stroma OP9 cells were cultured in α-MEM + 20% FCS. CD34^+^CD38^-^ cells were isolated from the cell lines as previously described^12, 24^. Briefly, the cells were labeled with monoclonal phycoerythrin (PE)-conjugated mouse anti-human CD34 IgG1, and allophycocyanin (APC)-conjugated mouse anti-human CD38 (all antibodies purchased from BD Biosciences, San Jose, CA, USA).

### Isolation of CD34^+^CD38^-^ALDH^int^ leukemia stem cells

Clinical bone marrow samples were obtained from patients with newly-diagnosed t(8;21) CBF AML granting informed consent as approved by the Johns Hopkins Medical Institutes' Institutional Review Board. The Johns Hopkins Institutional Review Board has approved these studies. CD34^+^ cells were isolated as we have previously described[25, 28]. Briefly, mononuclear cells will be isolated from fresh samples by Ficoll-Paque (GE Healthcare Life Sciences, Piscataway, NJ, density = 1.077) centrifugation. CD34^+^ cells were selected by MiltenyiBiotec (Auburn, CA, USA) microbeads (binding the class II CD34 epitope) and column per manufacturer's instructions and cryopreserved until further use. The thawed CD34^+^ cells were labeled with CD34 and CD38 as described above, and then stained with Aldefluor (Aldagen, Durham, NC) per manufacturer's guidelines. The CD34^+^CD38^-^ALDH^int^ cells were then isolated using a FACSAria (BD Biosciences), and cultured in RPMI1640 supplemented with 10% FBS (Sigma-Aldrich), 100 μg/mL penicillin-streptomycin (P/S; Sigma), and growth factors [thrombopoietin 20 ng/mL, Stem Cell Factor 100 ng/mL, and Flt3 ligand 100 ng/mL (TSF) (all growth factors and cytokines are from Amgen)], and incubated at 37°C.

### Isolation of primary bone marrow stroma

Primary bone marrow stroma cells were derived from normal bone marrow donors granting informed consent as approved by the Johns Hopkins Medical Institutes' Institutional Review Board, as we have previously described[25]. Briefly, mononuclear cells isolated from bone marrow of normal volunteers were cultured in FBMD1 media [IMDM media (Gibco) supplemented with 15% FBS (Sigma-Aldrich), 5% Horse serum (Sigma-Aldrich), 100 μg/mL penicillin-streptomycin (Gibco), and 10^-4^ M β-mercaptoethanol (Sigma-Aldrich)][29] at 33°C in 5%CO_2_ overnight. The next day, media and cells in suspension were removed and the attached cells were washed twice with phosphate-buffered saline (PBS) (Gibco), fresh FBMD1 media was added to the flask and they were placed back 33°C in 5%CO2. Half of the media was replaced weekly until an adherent monolayer has formed. At that time, the cells were dissociated using Trypsin (Gibco) and they were either used for further experiments or cryopreserved. The passage number of the cells was recorded with original cells labeled as P1. Experiments presented in this paper were performed using bone marrow stroma at passages 2–4.

### Co-culture system

For co-culture conditions, 24-well plates (Sigma) were coated with 0.1% Gelatin (Sigma) in PBS for 30 min. at 37°C. Gelatin solution was removed and stroma cells were seeded at a density of 5x10^4^ cells/well and cultured until a confluent monolayer was obtained. Subsequently, 2.5x10^4^ NB4, and OCI/AML3 cells and 5x10^4^ Kasumi-1 cells and primary patient LSCs were plated per well. The cultures were treated with or without 10^-7^M RA (for NB4 cells) or 10^-6^M RA (for all other AML cells) as well as 10^-6^M R115866 (CYP26 inhibitor—generously supplied by Johnson & Johnson, R&D) for 72h.

### Colony forming unit (CFU-C)

Clonogenic growth of AML cell lines was evaluated as we previously described[30, 31]. Briefly, previously treated cells were removed from the plate and washed with PBS to remove the respective drug. Cells were then counted using Trypan blue and plated 1 mL 1.2% methylcellulose (Sigma-Aldrich), 30% bovine serum albumin (BSA) (Sigma-Aldrich), 10^-4^ M β-mercaptoethanol (Sigma-Aldrich), and 2 mM L-glutamine (Gibco). Samples were plated in triplicate onto 35-mm^2^ tissue culture dishes and incubated in a humidified atmosphere at 37°C and 5% CO_2_. Colonies consisting of more than 40 cells were scored at 5–10 days using an inverted microscope.

### Flow cytometry

The cell lines and clinical AML samples were analyzed for expression of cell surface antigens using FACS Calibur (BD Biosciences, CA, USA). Briefly, AML cells were washed with PBS containing 0.2% BSA and stained with the following antibodies for 30 min at 4°C: phycoerythrin (PE)-conjugated mouse anti-human CD11b IgG1, fluorescein isothiocyanate (FITC)-conjugated mouse anti-human CD15 IgM antibodies, FITC-conjugated mouse anti-human CD34 IgG1, PE-conjugated mouse anti-human CD38 IgG2α, and allophycocyanin (APC)-conjugated mouse anti-human CD45 IgG2β antibodies or their respective isotype controls. All antibodies were purchased from BD Biosciences. Cells will be then washed to remove unbound antibody, and evaluated using a FACS Calibur (BD Biosciences) with a minimum acquisition of 10,000 events.

### Retinoic acid quantification

Culture media (RPMI+10%FCS), supplemented with 10^-6^M atRA was incubated at 37°C, 5% CO_2_ humidified tissue culture incubator in the presence or absence of OP-9 bone marrow stroma with or without 10^-6^M R115866. Media was harvested after 0h, 2h, 8h and 24h, was spun down to eliminate any potential cellular debris and was frozen and stored at -80°C until analysis. For analysis, media was extracted with two step liquid-liquid extraction as previously described[32]. RA isomers were quantified using LC-MS/MS on a AB Sciex 5500 QTRAP in MRM mode using APCI in positive ion mode as previously described[32, 33].

### Statistical analysis

Statistical analysis was performed by using two-tail unpaired student t test to compare the averages of two groups and calculate the p value.

## Results

### Stromal CYP26 prevents ATRA-induced differentiation of APL cells

**Fig 1:**
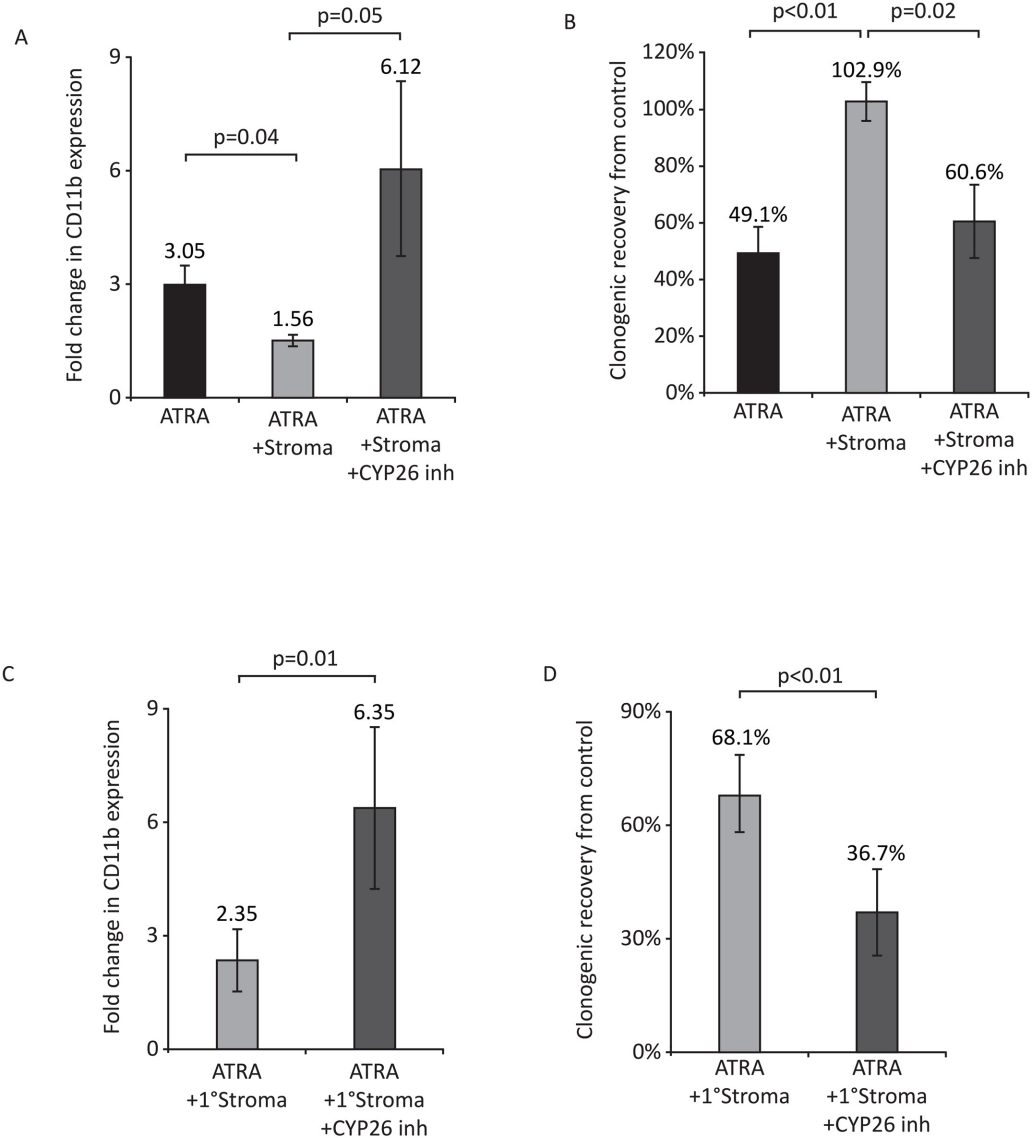
Effects of BM stroma on atRA-induced differentiation of APL cells. Effects of 10^-7^M ATRA±10^-6^M R115866 for 72h on the (A) expression of CD11b and (B) clonogenic growth of NB4 APL cells in the presence of OP9 BM stroma. Data represent mean ± SEM of three independent experiments. Effects of 10^-7^M atRA ± 10^-6^M R115866 for 72 hours on the (C) expression of CD11b and (D) clonogenic growth of NB4 APL cells in the presence of primary human BM stroma from normal donors. ATRA only controls for these conditions are presented in panels A and B respectively. Results represent mean ± SEM of independent experiments using four individual primary stromas. Treatment of APL cells with CYP26 inhibitor with or without ATRA in the absence of stroma or in the presence of stroma without ATRA had no effect on differentiation status of these cells.

It is well documented that atRA differentiates NB4 APL cells with subsequent decreased cellular expansion (S1A Fig), cell cycle arrest (S1B Fig), up-regulation of differentiation markers (S1C Fig), decreased blasts (S1D and S1E Fig) and clonogenic loss (S1F Fig). We recently showed that BM mesenchymal stroma protects normal HSCs from atRA-mediated differentiation through the expression of CYP26, the major mechanism of retinoid inactivation[25]. NB4 co-cultures with the mouse BM stromal line OP9 similarly blocked atRA (10^-7^M atRA for 72h) mediated induction of CD11b expression and clonogenic loss (Fig 1A and 1B). Inhibition of CYP26 activity via R115866 restored atRA-induced up regulation of CD11b and inhibition of clonogenic activity. The addition of the CYP26 inhibitor in the absence of stroma had no effect on atRA-induced differentiation (data not shown). Primary human BM stroma also protected against atRA-induced differentiation of NB4 cells. Low passage (<P3) human primary BM stromal cultures from four normal volunteers were co-cultured with NB4 APL cells and atRA. As with the OP9 stromal cells, primary stroma blocked atRA-induced differentiation that was rescued by CYP26 inhibition (Fig 1C and 1D). Consistent with the lack of direct cytotoxic effects of ATRA on APL cells, exposure of these cells to retinoids for 72h in all the conditions analyzed resulted in no significant differences in cellular numbers.

### Stromal CYP26 metabolizes atRA

**Fig 2:**
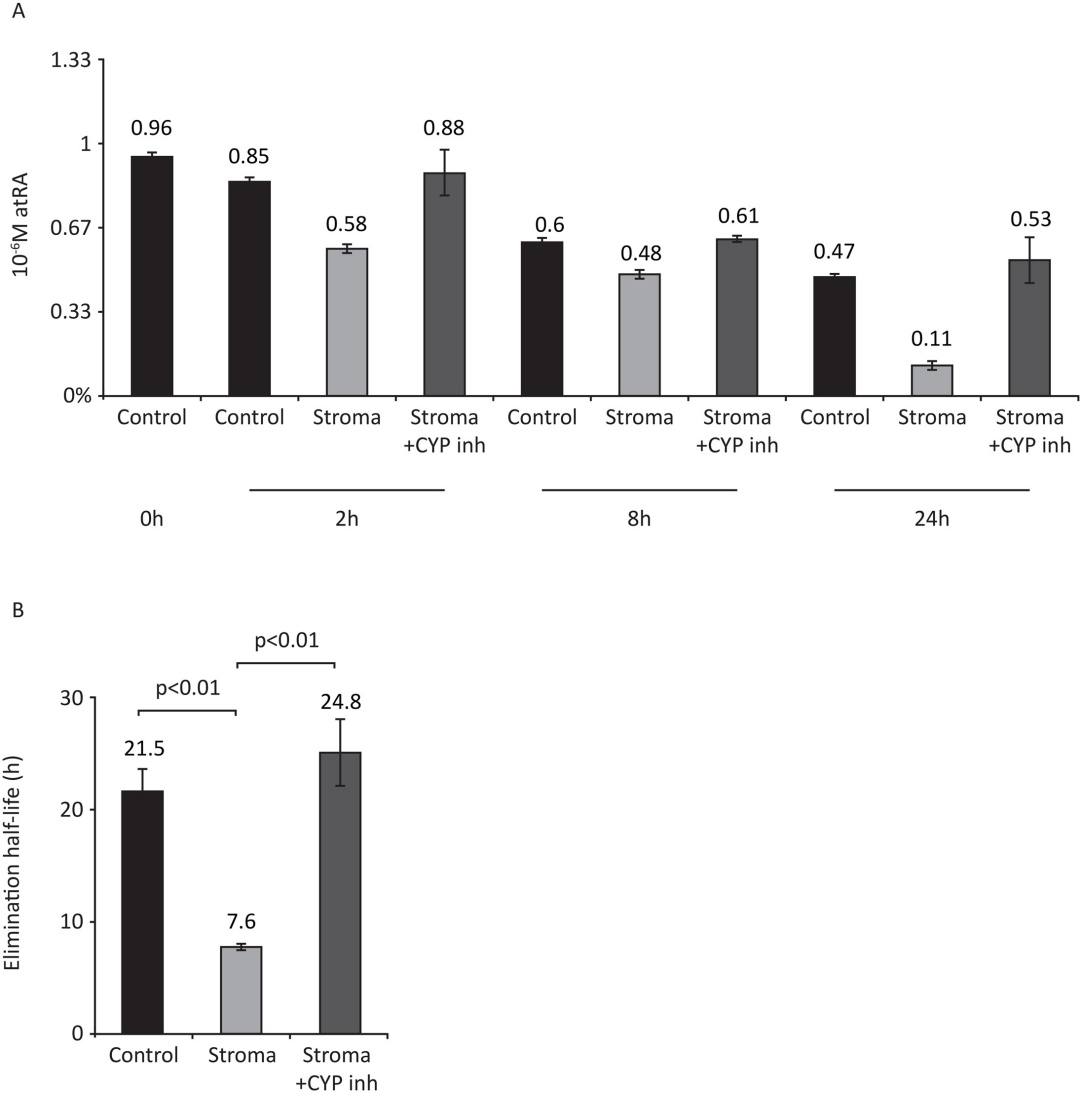
Effect of BM stroma on atRA concentrations. Media (RPMI+10%FCS) was supplemented with 10^-6^M atRA and incubated at 37°C in a humidified incubator in 5%CO_2_ either in the absence of stroma (Control) or in the presence of stroma with or without 10^-6^M CYP26 inhibitor (R115866). A) The concentration of atRA was determined at 0h, 2h, 8h and 24h. B) Elimination half-life of atRA (t_1/2_ = 0.693/slope) calculated from the natural logarithms of percent atRA remaining over time. The half-life of atRA during culture conditions was 21.5±1.9h in the absence of stroma (Control), 7.6±0.4h in the presence of stroma and 24.8±3.2h in stroma+CYP inhibitor conditions. Data represent mean ± STD of three independent experiments.

To prove that stromal CYP26 can metabolize pharmacological levels of atRA, BM stroma was incubated with 10^-6^M atRA, and atRA was quantified in the conditioned medium by high pressure liquid chromatography—tandem mass spectrometry (HPLC-MS/MS) (Fig 2A and 2B). In the presence of BM stroma, there is a time dependent decrease in atRA levels such that at 24h only about 10% of the atRA remained (p<0.01). Inhibition of CYP26 by R115866 blocked the metabolism of atRA such that atRA levels were comparable to no stroma controls (p = 0.37) (Fig 2A). Consistent with previous reports[34], the elimination half-life of atRA in the absence of stroma was 21.5±1.9h; the presence of BM stroma decreased the half-life to only 7.6±0.4h (p<0.01). CYP26 inhibitor rescued atRA half-life to levels comparable to no stroma control (24.8±3.2h, p = 0.34 vs. control).

### BM stroma blocks atRA-induced differentiation of non-APL AML via CYP26

**Fig 3:**
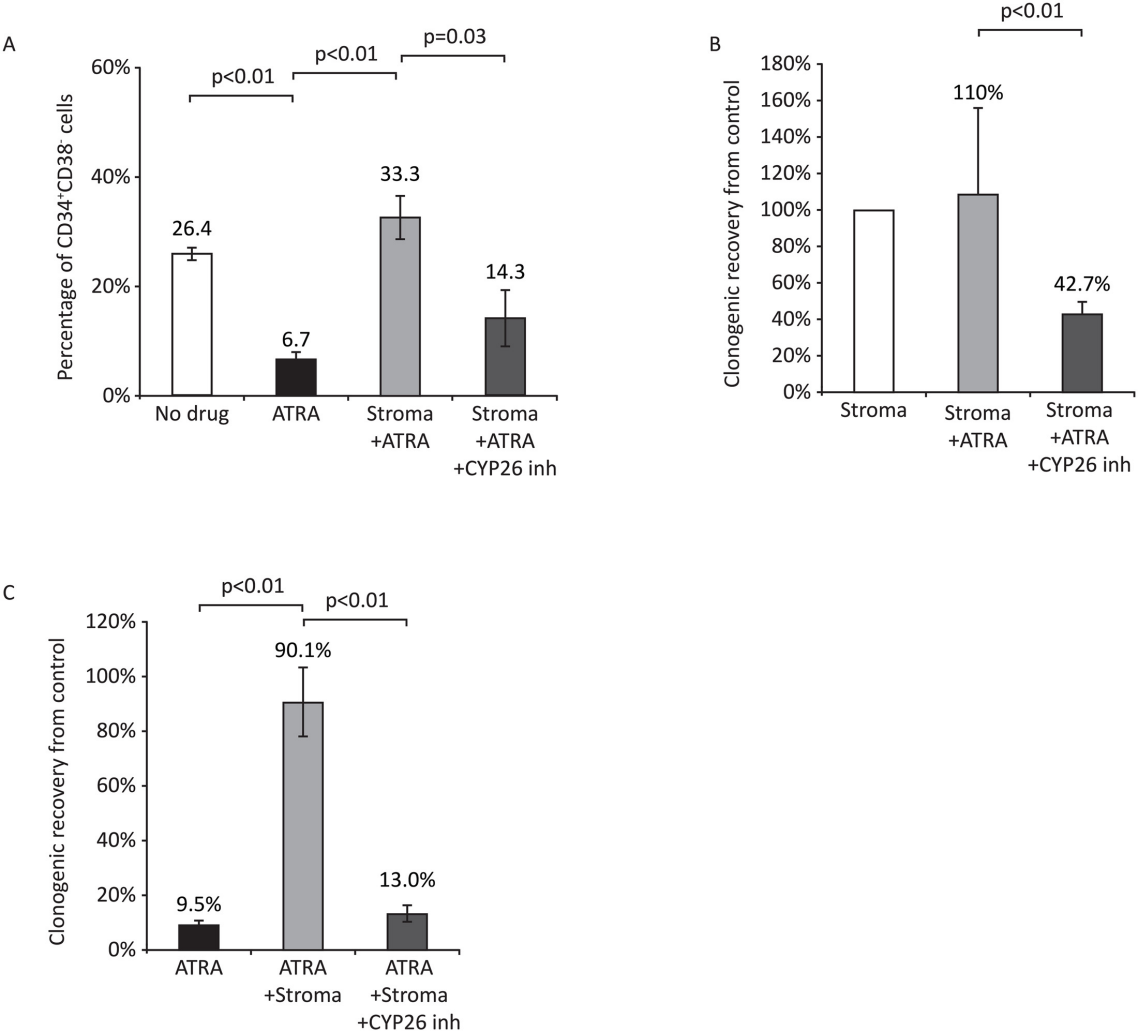
Effects of bone marrow stroma on atRA-induced differentiation of non-APL AML cells. Effects of 10^-6^M atRA ± 10^-6^M R115866 for 72 hours on (A) the phenotypic differentiation and (B) clonogenic growth of Kasumi-1 cells in the presence of OP9 bone marrow stroma. CD34^+^CD38^-^ Kasumi-1 cells were isolated by flow cytometry and cultured as described. Data are presented as mean ± SEM of four (A) or three (B) independent experiments respectively. C) Effects of 10^-6^M ATRA ± 10^-6^M R115866 for 72 hours atRA on the clonogenic growth of OCI/AML-3 cells in the presence of OP9 bone marrow stroma. Data represent the mean ± SEM of three independent experiments. Treatment of AML cells with CYP26 inhibitor with or without ATRA in the absence of stroma or in the presence of stroma without ATRA had no effect on differentiation status of these cells.

atRA has also been shown to induce differentiation of the t(8;21) core binding factor (CBF) AML cell line Kasumi-1[35], although it has shown no clinical activity in CBF AML[15–18]. About 20–30% of Kasumi-1 cells exhibit a HSC phenotype (CD34^+^CD38^-^) (S2 Fig), and culture in the presence of atRA resulted in rapid loss of CD34^+^CD38^-^ compartment both phenotypically and by clonogenic recovery (Fig 3A and 3B, p<0.01). Kasumi-1 CD34^+^CD38^-^ cells and their clonogenic activity were protected from atRA-induced differentiation when co-cultured with BM stroma (Fig 3A and 3B). This protection was rescued by inhibition of CYP26. Again, CYP26 inhibition had no effect in stromal-free cultures (data not shown).

While atRA has shown *in vitro* activity against NPM1-mutated AMLs[36], most studies[37, 38] have not confirmed the initial report suggesting clinical activity in this AML subtype[15]. Thus, we investigated whether the microenvironment may also play a role in these divergent findings regarding the effect of atRA in NPM1-mutated AML. NPM1 mutated AML cell line OCI-AML3, was treated with atRA in the absence or presence of BM stroma (Fig 3D). Treatment of OCI-AML3 cells with 1μM atRA for 72h resulted in 90% loss of clonogenic growth (p<0.01), while similar treatment in the presence of BM stroma had no effect on OCI-AML3 clonogenic recovery. Inhibition of CYP26 by R115866 overcame the protective effect of stroma against OCI-AML3 treated with atRA (p<0.01). Consistent with the lack of direct cytotoxic effects of ATRA on AML cells, exposure of these cells to retinoids for 72h in all the conditions analyzed resulted in no significant differences in cellular numbers.

### Primary leukemia stem cells (LSCs) are protected from atRA via niche CYP26

**Fig 4:**
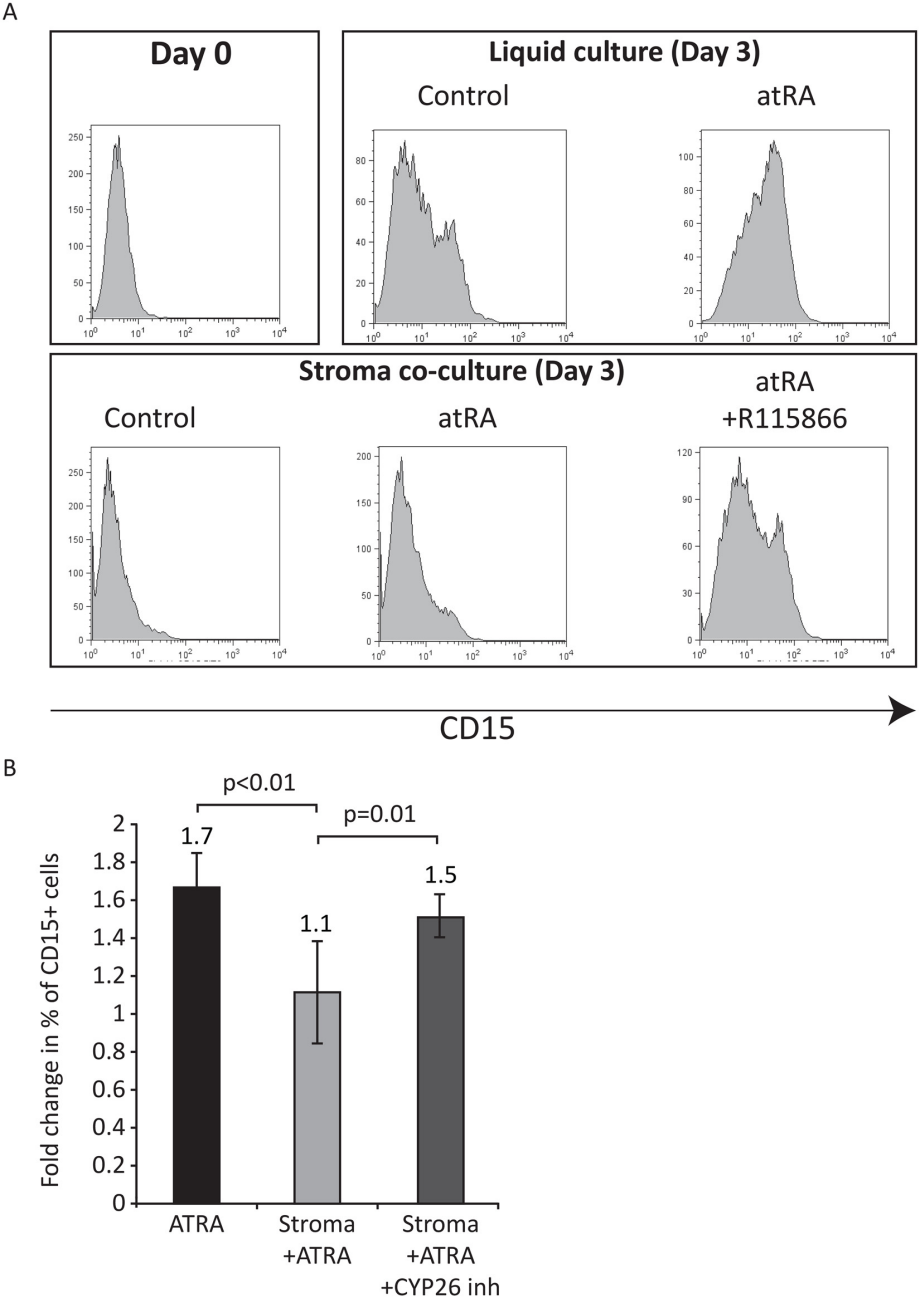
Bone marrow stromal effects on atRA-induced differentiation of primary CBF LSCs. (A) One representative experiment (from four independent patients with similar results) of CD15 expression of CBF AML CD34^+^CD38^-^ALDH^int^ cells prior to culture (left upper panel), post 72h of culture in the absence of BM stroma (right upper panel), or in the presence of BM stroma (lower panel). Prior to culture, few if any LSCs express CD15 (range 0.1%-3%, left upper panel). After culture for 72h in RPMI+10%FCS and thrombopoietin, kit ligand, and Flt3 ligand, a range 8.3%-41.7% of cells expressed CD15. (B) Quantitative results from all 4 experiments showing fold change of proportion of cells expressing CD15 from control cultures. Data are presented as mean ± SEM of fold change from control using CD34^+^CD38^-^ALDH^int^ cells from four different patients with CBF AML. Treatment of LSCs with CYP26 inhibitor with or without ATRA in the absence of stroma or in the presence of stroma without ATRA had no effect on differentiation status of these cells.

Laboratory data suggest that AML maintains the basic hierarchical structure of normal hematopoiesis; i.e., rare cells possessing self-renewal capacity, so-called LSCs, give rise to partially differentiated progeny that compose the tumor bulk but possess only limited proliferative potential[39]. Although the clinical significance of LSCs has been questioned, recent data strongly implicate LSCs (CD34^+^CD38^-^ intermediate ALDH activity or ALDH^int^) in disease relapse; MRD was enriched for LSCs, and their presence after therapy highly correlated with subsequent clinical relapse[28]. To test if BM microenvironment protects primary LSCs from atRA, we isolated CD34^+^CD38^-^ALDH^int^ cells from the BM of patients with newly-diagnosed CBF [t(8;21)] AML. Prior to culture, these cells expressed no differentiation markers such as CD15 (Fig 4A), CD33, and CD11b (data not shown). Culture of these cells in media containing 10% serum (and about 1nM atRA)[40] led to acquisition of differentiation markers including CD15 (Fig 4A—upper middle panel). The addition of 1μM atRA induced further up regulation of CD15 (Fig 4A; upper right panel, and 4B). Culture in the presence of BM stroma inhibited acquisition of CD15 (Fig 4A, lower middle panel, and 4B), and this was rescued by inhibition of stromal CYP26 (Fig 4A, lower right panel, and 4B, p = 0.01).

## Discussion

The early success seen with the introduction of atRA in treatment protocols of APL raised hope that differentiation therapy could change the face of current treatments in leukemia; this was further bolstered by evidence of atRA’s activity against most non-APL AMLs *in vitro*[7–12]. Unfortunately, initial enthusiasm has been dampened by largely negative results from clinical trials using combination atRA + chemotherapy for induction regimens in non-APL AML[16–18]. Nevertheless, preclinical data has shown that atRA was actually able to induce terminal differentiation of many subtypes of AML *in vitro*[41].

Our data suggest that the leukemic microenvironment could provide a potential explanation for the lack of clinical benefit of atRA despite *in vitro* activity, but few if any studies have actually focused on the effects of the microenvironment on atRA activity in AML. Results presented here show that even the most sensitive AML cells, including APL, become resistant to atRA treatment in the presence of stromal CYP26 activity. We previously showed that the stem cell niche CYP26 expression also protected normal HSCs from retinoids, helping to maintaining them in a quiescent, undifferentiated state[25]. Interestingly, since CYP26 is directly up-regulated by retinoids[42] in a variety of tissues including hepatocyte, intestine, endothelial cells and even leukemia[43], treatment with pharmacological doses of atRA could induce CYP26 expression by the BM niche and produce an even more protective microenvironment for LSCs. In-depth analysis of CYP26 levels in the BM microenvironment of patients with AML and how they change during therapy are warranted for further understanding of how the protective niche changes in patients with AML. Niche inactivation of atRA could also partially explain its ability to induce terminal differentiation of the malignant promyelocytes in APL, but the inability to eliminate LSCs.

The relative effectiveness of atRA in APL may result from increased intrinsic sensitivity of APL cells comparted to non-APL. The dose of atRA (1M) needed to inhibit non-APL cells in the absence of stroma is a log higher than the similar dose (0.1M) active in APL cells (Fig 4). Nevertheless, 1M is clinically achieved with therapeutic doses of atRA[44] suggesting there are also extrinsic reasons for the lack of effectiveness of atRA in non-APL AML. Disparities in differentiation status between APL LSCs and non-APL AML LSCs[45] may translate into occupancy of distinct niches which could contribute to the differential clinical activity seen with ATRA in APL.

Our results reveal therapeutic opportunities for improving the effectiveness of retinoids in AML by overcoming the microenvironment’s ability to inactivate atRA. Several CYP26 inhibitors, including R115866 used here, have safely been in clinical trials for other indications such as acne and psoriasis[46]. Systemic inhibition of CYP26 is expected to increase plasma atRA levels with potential increased toxicity. Thus, pharmacologically adjusting atRA doses to maintain safe systemic concentrations in the presence of CYP26 inhibition, should control for hepatic inhibition of the enzyme while at the same time removing the barrier to therapeutic atRA levels in the microenvironment. The synthetic retinoid tamibarotene (AM80) has activity in atRA-resistant APL and is approved in Japan for this indication[47]. The resistance of tamibarotene to CYP26[48] may be responsible for its activity in atRA-resistant APL. Such approaches that circumvent CYP26 in the leukemic microenvironment could expand the effectiveness of retinoid-based therapy in both APL and non-APL AML.

## Supporting Information

S1 Fig Effects of ATRA on NB4 APL cells. A) Cellular expansion of NB4 APL cells in the presence of 10-7M ATRA. ATRA treated cells (triangles) expand 3.4±0.5 fold by Day (D)3 of cultures compared to 4±0.3 fold in control culture (p = 0.14). Continued exposure to ATRA for 6 days results in 3.3±1.6 fold expansion from D0 compared to 32.7±1.3 in control cultures (p<0.01). Data represent mean ± SEM of three independent experiments. B) Cell cycle analysis of NB4APL cells treated with ATRA for 6 days. One representative experiment from three with similar results show increased cells in G0/G1 phases of cell cycle upon treatment with ATRA C) Expression CD11b, a differentiation marker of NB4 APL cells. Upon culture in the presence of ATRA for 72h the mean fluorescence intensity (MFI) of CD11b expression of NB4 cells was 3.05±0.48 fold higher compared to control cultures. Data represent mean ± SEM of three independent experiments, p = 0.01. D) Effects of prolonged ATRA treatment on morphologically defined NB4 APL blasts. ATRA treated cultures have 24.2%±3.8% blasts at D6 compared to 44.5%±7.1% in control cultures. Data represent mean ± STD of four independent experiments, p<0.01.E) Effects of ATRA treatment for 6 days on cell size (indicated by Forward Scatter) and cytoplasmic complexity (indicated by right angle Side Scatter). Compared to control (red circle), ATRA treated cultures contain a population of cells (blue oval) that are relatively smaller and have increased cytoplasmic complexity. One representative experiment from three with similar results. F) Effects of 10-7M ATRA on the clonogenic activity of NB4 APL cells. Treatment with ATRA for 72h results in 49.1%±8.9% clonogenic recovery from control cultures. Data represent mean±SEM of eight independent experiments, p<0.01.
(PDF)

S2 Fig Flow cytometry analysis of Kasumi-1 cells. Expression of CD34 and CD38 prior to sort (upper left plot) and post sort but prior to further culture (upper right panel). Middle and lower panels show CD34+CD38- sorted Kasumi-1 cells after 72h of culture in the absence (Liquid culture) or presence (Stroma co-culture) of BM stroma, respectively. Cells treated with 1μM atRA are presented in middle right panel and lower mid panel, and cells treated with 1μM atRA+1μM CYP26 inhibitor, R115866 are presented in lower right panel. Dead cells were gated out based on 7AAD positivity and non-hematopoietic cells (i.e. BM stroma) were dismissed based on lack of CD45 expression. This is one representative experiment from four with similar results.
(PDF)
