# Supplementary figures and images for: All-Trans Retinoic Acid Activity in Acute Myeloid Leukemia: Role of Cytochrome P450 Enzyme Expression by the Microenvironment
Source: PLoS One. 2015 Jun 5;10(6):e0127790. doi: 10.1371/journal.pone.0127790 (PMC4457893; doi:10.1371/journal.pone.0127790)

Supplementary Figure 1

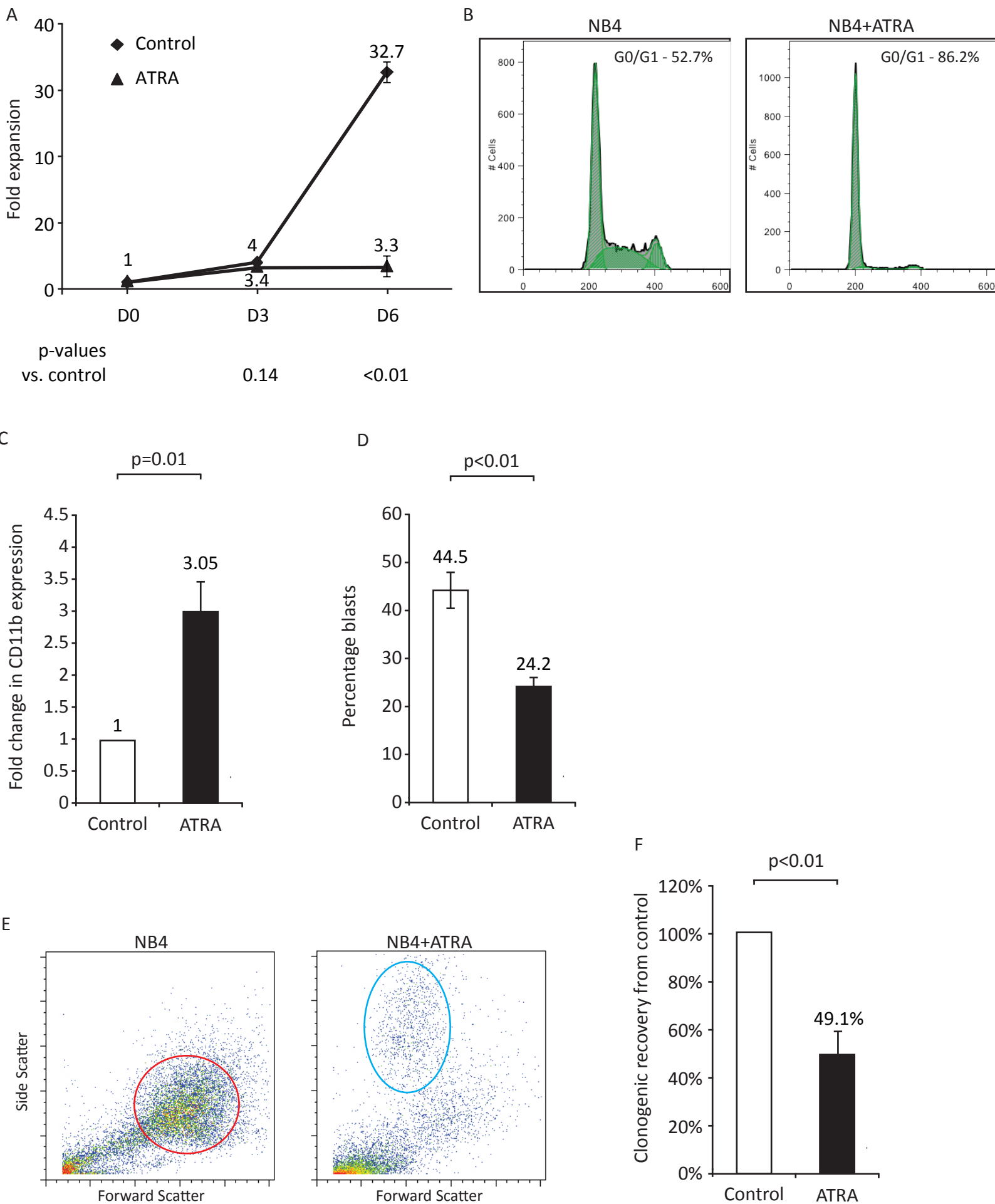

Supplement: S1 Fig — A) Cellular expansion of NB4 APL cells in the presence of 10-7M ATRA. ATRA treated cells (triangles) expand 3.4±0.5 fold by Day (D)3 of cultures compared to 4±0.3 fold in control culture (p = 0.14). Continued exposure to ATRA for 6 days results in 3.3±1.6 fold expansion from D0 compared to 32.7±1.3 in control cultures (p<0.01). Data represent mean ± SEM of three independent experiments. B) Cell cycle analysis of NB4APL cells treated with ATRA for 6 days. One representative experiment from three with similar results show increased cells in G0/G1 phases of cell cycle upon treatment with ATRA C) Expression CD11b, a differentiation marker of NB4 APL cells. Upon culture in the presence of ATRA for 72h the mean fluorescence intensity (MFI) of CD11b expression of NB4 cells was 3.05±0.48 fold higher compared to control cultures. Data represent mean ± SEM of three independent experiments, p = 0.01. D) Effects of prolonged ATRA treatment on morphologically defined NB4 APL blasts. ATRA treated cultures have 24.2%±3.8% blasts at D6 compared to 44.5%±7.1% in control cultures. Data represent mean ± STD of four independent experiments, p<0.01.E) Effects of ATRA treatment for 6 days on cell size (indicated by Forward Scatter) and cytoplasmic complexity (indicated by right angle Side Scatter). Compared to control (red circle), ATRA treated cultures contain a population of cells (blue oval) that are relatively smaller and have increased cytoplasmic complexity. One representative experiment from three with similar results. F) Effects of 10-7M ATRA on the clonogenic activity of NB4 APL cells. Treatment with ATRA for 72h results in 49.1%±8.9% clonogenic recovery from control cultures. Data represent mean±SEM of eight independent experiments, p<0.01. (PDF) [file pone.0127790.s001.pdf]

Supplementary Figure 2

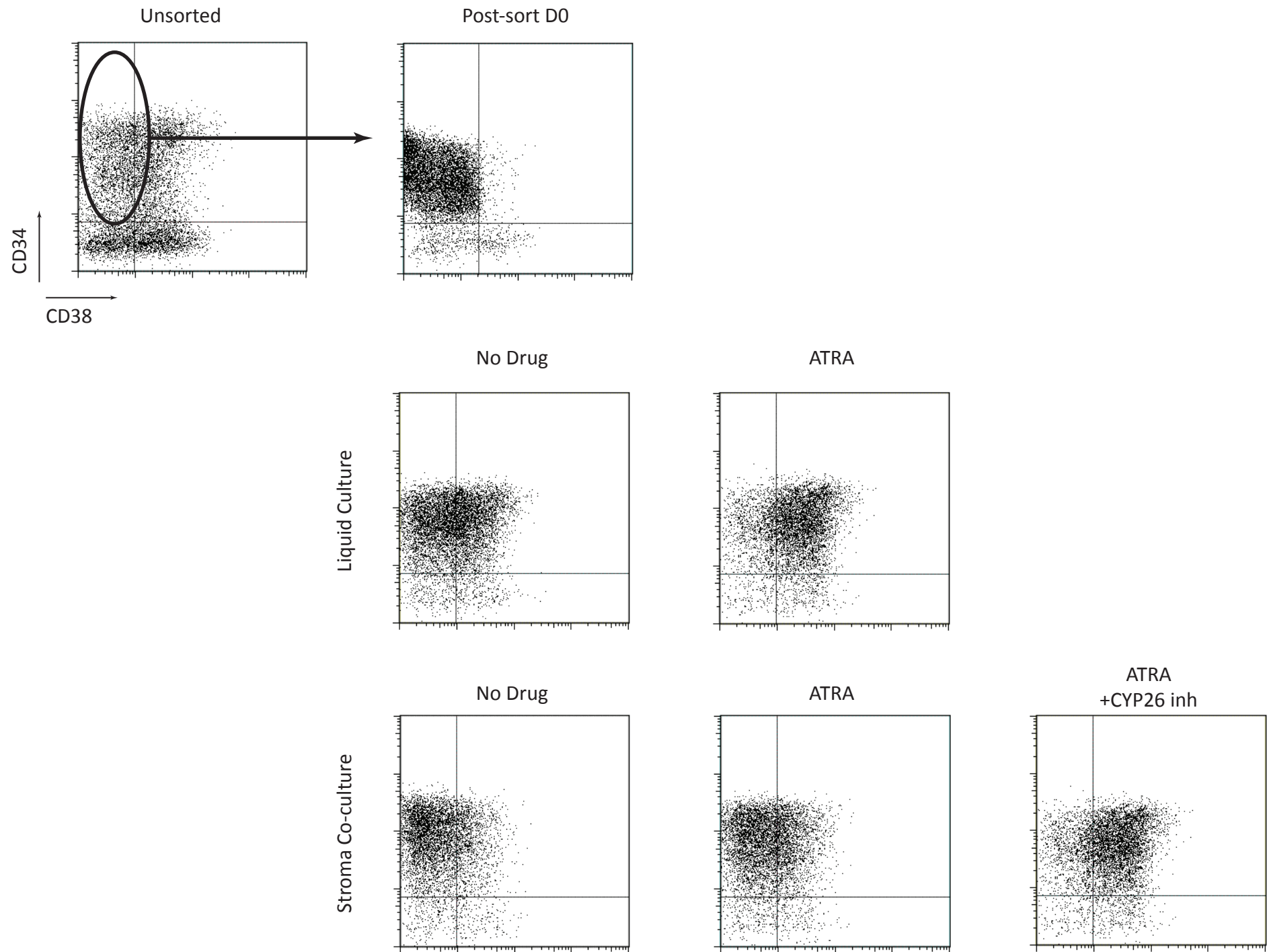

Supplement: S2 Fig — Expression of CD34 and CD38 prior to sort (upper left plot) and post sort but prior to further culture (upper right panel). Middle and lower panels show CD34+CD38- sorted Kasumi-1 cells after 72h of culture in the absence (Liquid culture) or presence (Stroma co-culture) of BM stroma, respectively. Cells treated with 1μM atRA are presented in middle right panel and lower mid panel, and cells treated with 1μM atRA+1μM CYP26 inhibitor, R115866 are presented in lower right panel. Dead cells were gated out based on 7AAD positivity and non-hematopoietic cells (i.e. BM stroma) were dismissed based on lack of CD45 expression. This is one representative experiment from four with similar results. (PDF) [file pone.0127790.s002.pdf]
